# Implementing “Chest Pain Pathway” Using Smartphone Messaging Application “WhatsApp” as a Corrective Action Plan to Improve Ischemia Time in “ST-Elevation Myocardial Infarction” in Primary PCI Capable Center “WhatsApp-STEMI Trial”

**DOI:** 10.1097/HPC.0000000000000264

**Published:** 2021-11-23

**Authors:** Wesam A. Alhejily

**Affiliations:** From the * Department of Medicine, Division of Cardiology, Faculty of Medicine, King Abdelaziz University Hospital, Jeddah, Saudi Arabia; † Cardiology Division, Dr Sulaiman Alhabib Medical Group, Saudi Arabia.

**Keywords:** acute coronary syndrome, ST-elevation myocardial infarction, door-to-needle time, chest pain, clinical pathway, improvement pathway

## Abstract

**Background:**

ST-elevation myocardial infarction (STEMI) is a life-threatening medical emergency that requires immediate medical attention. Each hospital should implement a clinical pathway with the main objective to reduce ischemia time from diagnosis to revascularization (because this has shown to save myocardial tissues and, subsequently, patients’ lives), utilizing the most evidence-based approach and the most up-to-date management protocol. In this study, we aimed to assess the utility of structuring chest pain pathway (CPP) with the instantaneous case-by-case feedback protocol using WhatsApp and its impact on improving 2 major key performance indicators—the impact on initial emergency room (ER) door-to-electrocardiogram (ECG) time, and door-to-balloon time—in a predefined 6-month corrective action period.

**Methods:**

A prospective, quality improvement plan was set to reduce door-to-first-ECG and door-to-balloon times to less than 10 and 90 minutes, respectively, in all acute coronary syndrome cases (including STEMI cases), from August 2020 to April 30, 2021. Several measures were done to attain the goal, including strict compliance to CPP with documentation, ruling out possible mimickers, and reducing false activation and time delays in ER. Direct feedback on cases was provided using WhatsApp messaging app on smartphones; protocol and results of time lines were accessed by all healthcare providers associated with CPP, including ER nurses, ER doctors, Cardiology residents, specialists, Catheterization laboratory nurses, technicians, and interventionalists. Bimonthly meetings with all stake holders were reinforced, and minutes of meeting were reviewed and corrective actions were implemented the next day. On a daily basis, all cases were analyzed and tabulated by a dedicated nurse and a doctor from ER, and another nurse and doctor from the quality assurance department.

**Results:**

After a 6-month period of implementing improvement plan and sticking to a comprehensive chest pain clinical pathways strategy with a case-by-case review on a shared smartphone messaging application, the rate of door-to-first-ECG improved from 76% to 93% in patients with chest pain, and the door-to-balloon time targeting less than 90 minutes improved significantly from only 77% to 92% in STEMI patients. This was statistically significant with a *P* value of 0.0001 and 0.001, respectively, and the rate of false activation was reduced from 23% to 16% and 10% 3 and 6 months of time, respectively, with a significant *P* value of <0.01. Finally, a strict 100% rate of timely documentation on patients with chest pain was achieved.

**Conclusions:**

An improvement plan of implementing the CPP, cut-time policy, and using smartphone WhatsApp messaging for a case-to-case instantaneous feedback has significantly improved key performance indicators and, subsequently, ischemia time in acute coronary syndrome patients (including STEMI patients) in 6 months time, indicating that this strategy works.

## Abstract 2

Supplemental Digital Content is available in the text.

## INTRODUCTION

Cardiovascular diseases (CVDs) remain the leading cause of death globally; more than 17.5 million people die from CVDs each year, representing 31% of all global deaths. Of the 31% global deaths, more than 25% are related to ST-elevation myocardial infarction (STEMI). The challenges to reduce time to revascularization are inevitable and exist even in the most experienced centers with primary percutaneous coronary intervention facility,^1^ and several guidelines were written to address the optimum care of these patients.^2,3^

Door-to-electrocardiogram (ECG) times and door-to-balloon time are 2 major key performance indicators (KPIs) in acute coronary syndrome (ACS) patients and more so for STEMI,^4–11^ with both proved to reduce time to revascularization and, subsequently, mortality. Having a dedicated ECG machine and a nurse for chest pains in triage improves ischemia time by improving triage education, disposition, and data feedback mechanism.^12,13^ Chest pain is a common challenging clinical presentation to emergency rooms: some patients may not have typical symptoms, others may not have initial diagnostic ECGs, and few patients may have an ST-elevation mimicker that looks like STEMI but could be related to other serious conditions like aortic dissection or pulmonary embolism. Therefore, it is advised that each hospital should implement a clinical pathway and an algorithm to help every healthcare provider envision the steps of diagnosing and initiating necessary treatment in such cases, in addition to teaching and continuous learning from cases. It is also important to have a continuous surveillance on hard outcomes related to death in patients with STEMI. The growing need for more consistency in patient care, improved resource efficiency, reduced costs, and improvements in the quality of patient healthcare have led to the development of chest pain pathways (CPPs). CPPs provide a structured timeline of actions needed to meet the goals of identifying high-risk ACS patients with improved efficiency when compared with traditional care. CPPs aim to improve quality and safety of patient care for this potentially life-threatening diagnosis, which ultimately reduce the risk of cardiac morbidity and mortality due to delayed treatment or inappropriate discharge. Additionally, it can improve resources utilization like reduction of length of stay and unnecessary hospital admissions.^14–20^

Constant feedback with a closed-loop debriefing for each case using different means of communication is known to improve outcomes.^21–28^ The use of WhatsApp (a smartphone messaging application) was already reported in the literature for quality improvement in multiple studies for telemedicine, emergency room disposition, and guidelines compliance. Herzog E. Elbaz et al^29^ used Pulmonary Embolism Response Team “PERT” in acute pulmonary embolism to bring together specialists from different disciplines. The team convenes in real time via a platform such as WhatsApp or text messages to communicate clinical data, discuss the options, and provide consensus for a course of management.^30–35^ Apart from these studies, no particular study in acute STEMI or chest pains pathway was identified in the literature.^30–49^

## METHODS

**FIGURE 1.**
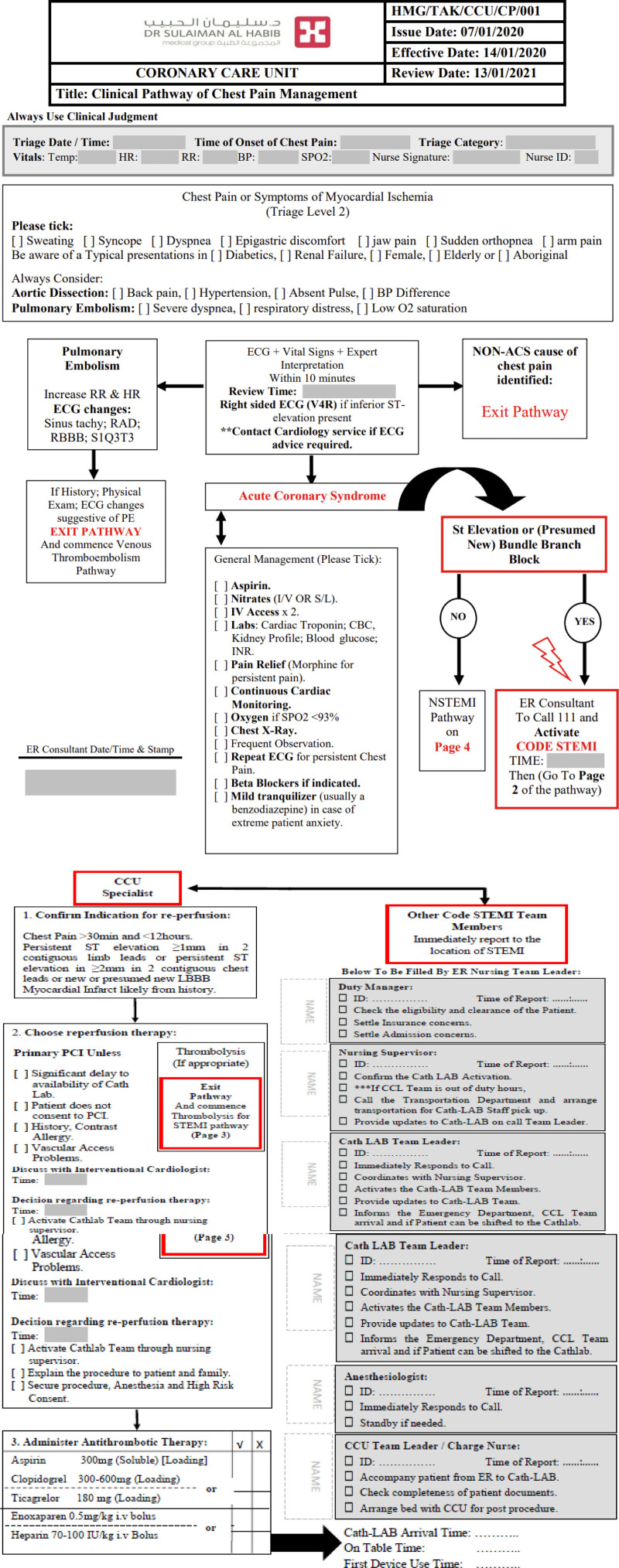
Chest pain pathway algorithm of acute coronary syndrome.

We adopted several bodies’ guidance when designing our clinical pathway for acute CPPs, including European and American Heart Association in addition to local and international expert bodies such as Central Board of Accreditation of Healthcare Institution and Joint Commission Association of Healthcare Organization.^2,3^ Two pages of the pathway are depicted in Figure 1, and the whole detailed pathway is enclosed in the Appendix, available at http://links.lww.com/HPC/A234. We follow the primary percutaneous coronary intervention protocol for all patients with STEMI, but the thrombolytic protocol is also depicted as an alternative approach in our algorithm. In rare occasions where Catheterization laboratory teams are busy with other STEMI cases, the thrombolytic protocol may be considered. We also teach Sgarbossa’s criteria for myocardial infarction in left bundle branch block; however, if the emergency room (ER) doctor is in doubt, it is always safe to activate “Code STEMI.”

The clinical pathway was approved by the department head, then by the medical executive committee, in January 2020 and reviewed biannually for new updates or points of care that need adjustments. Several meetings were held with cardiology, ER nursing, catheterization laboratory staff, CCU, and telemetry team as well as attending in cardiovascular department and hospital administration staff to confirm the algorithm. We reviewed the compliance statistics on a quarterly basis. The outcome of each case was discussed in a departmental meeting in the presence of the medical director, and cardiology and ER head of departments to evaluate the process and the clinical pathway implementation. On March 1, 2020, we conducted a prospective study looking at the impact of this stringent protocol dealing with chest pains and, more specifically, STEMI cases. The primary end point was “improvement in door-to-ECG time and door-to-balloon time.” The secondary end point was compliance rate and false activation. Compliance to this clinical pathway has been monitored through an auditing team on a quarterly basis. Real-time documentation and investigation for compliance to timeline of the pathway was ensured. We also established a “WhatsApp” group named “STEMI fast Track” to review all cases daily with instantaneous feedback and corrective action for any unexpected delays or mishandling of any cases. Patients’ personal information and demographic features were not posted on WhatsApp, in compliance with Health Insurance Patients Portability and Accountability act “HIPPA.” A dedicated Nurse in ER with a specified ECG machine was assigned for chest pain cases each shift, timeline of cases was charted in a shared folder in ER, ECG training for ER staff was conducted on several times during the study, code STEMI activation was done by ER consultants, with a single dedicated number at the switch board calling all members of Catheterization laboratory team at the same time. Since both primary endpoints are continuous variables and they depend on each other, we choose paired *t* test for analysis, assuming normally distributed data with 95% confidence intervals. The total period of the study is 9 months, 3 months at baseline with no intervention, and 6 months with improvement plan in place.

## RESULTS

Among 2067 patients visited the ER with chest pains in a period of 9 months, 457 patients were labeled as ACS patients, of which 63 patients had STEMI. The mean age was 58 ± 15, and 70% of the patients were men. Diabetic patients were 45%, and Hypertension was the diagnosis in 62.7%, hyperlipidemia was present in 39%, and positive family history was found in 11.6% of the population.

**FIGURE 2.**
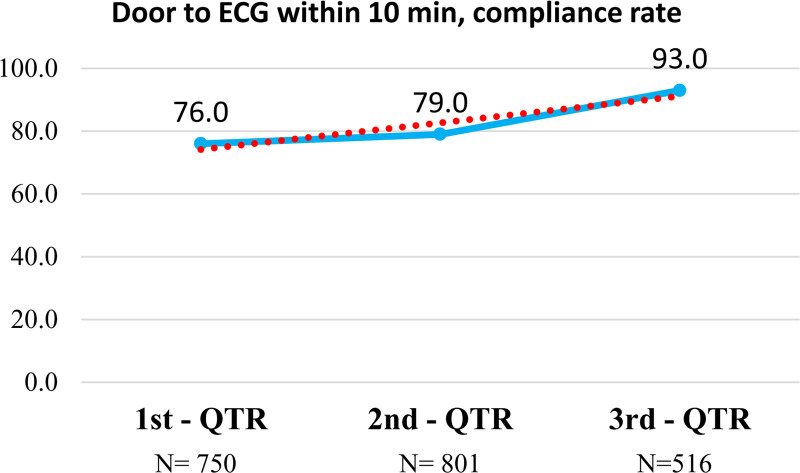
Compliance rate of door-To-ECG time within 10 minutes, N = total numbers of patients.

**FIGURE 3.**
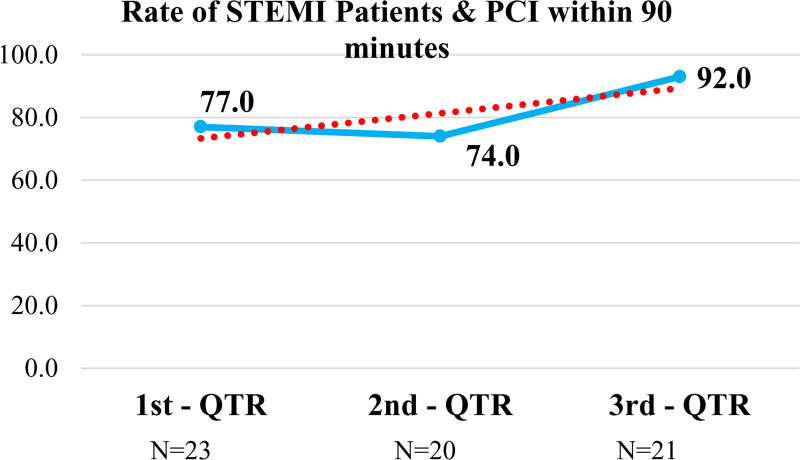
Rate of STEMI patients with door-to-needle time less than 90 minutes, N = Total number of patients.

**FIGURE 4.**
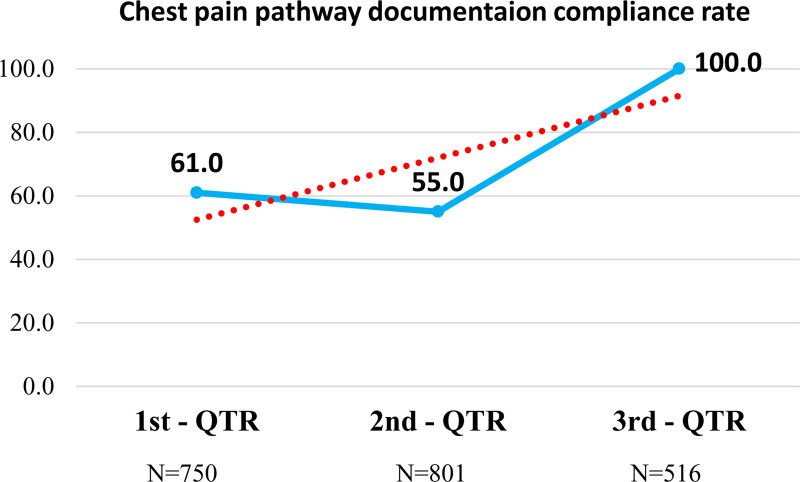
Chest pain pathway documentation compliance rate, N = Total number of patients.

**FIGURE 5.**
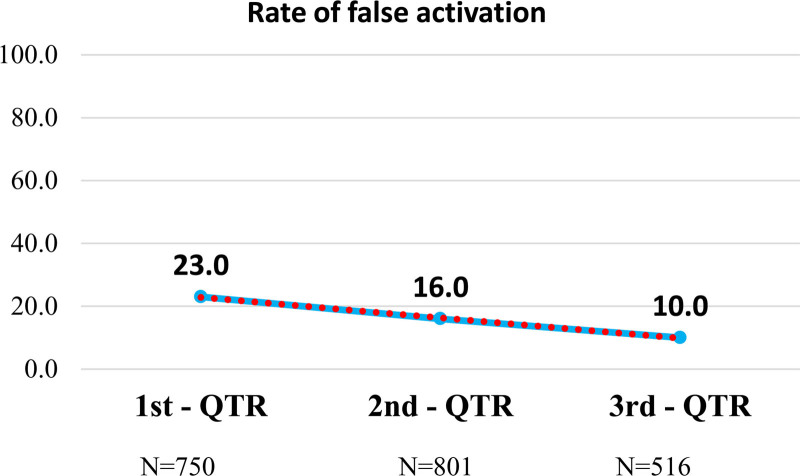
Rate of false activation among patients with chest pains, N = total number of patients.

The primary end point of achieving door-to-balloon time was improved after 6 months but not after 3 months from 77% to 74% to 92%, respectively, which was statistically significant with a *P* value of 0.001. It is important to highlight that the total study period is 9 months with quarter 1 is baseline with no protocol implemented, quarter 2 and 3 are under stringent improvement protocol implementation, the door-to-ECG time was reduced from 12 ± 3 minutes in the first quarter to 7 ± 3 minutes in the last quarter, and in 93%, when compared with 76% patients before the improvement program, this was significant after 6 months from starting the protocol (*P* = 0.0001). The secondary endpoints of compliance and rate of false activation was 100% and 10%, respectively. For the remaining 457 ACS patients, an early invasive strategy and early discharge plan were ensued with a median admission time of 2.5 days compared with 4 days length of stay before the improvement program; appropriate discharge medications (including aspirin, beta blockades, statins, and ACE inhibitors) were all improved after 6-month interval (Figs. 2–5).

## DISCUSSION

Chest pain remains one of the most challenging presentations in any emergency room because some causes of chest pain carry a risk to patient’s lives. The ability to separate life-threatening from non-life-threatening causes is a crucial step in making a correct diagnosis and, subsequently, proper management plans. While most hospitals have CPPs, compliance rate remains low. In this study, we opted to evaluate the impact of improving compliance rate of implementing CPP and the effect on benchmarks like door-to-balloon and door-to-ECG time.

This is a real-time study that reflects the impact of implementing a comprehensive CPP with continuous surveillance program on patients presented to ER with suspected ACS. We launched a “WhatsApp” group for an instantaneous feedback for every chest pain that is thought to be ACS and, more importantly, when an ST-elevation MI was the final diagnosis. Cases were posted on the group once activation is made and timeline is provided at the end of each case and commented on by the team leaders. We also made a weekly assessment of all CPPs to ensure that clinical pathway was implemented, forms were filled, timeline was accurately reflected in the clinical pathway, direct feedback was given, and areas of improvement were highlighted. After conducting the program, we noticed no significant changes in the first 3 months as we were just learning causes of delays and time lost in the pathway. However, in the third quarter (after 6 months of implementing the protocol), there was a significant improvement in benchmarks of door-to-balloon time and chest pain to first ECG, both of which correlated well with strict compliance to clinical pathway of chest pains in the emergency room. One of the major reasons of delays we identified is delay related to first ECG in more than 35% of the time either due to a lack of proper history taking at initial triage or because the ECG machine is not readily available. The second cause of delay was the lack of communication between triage nurse and the ER physician after initial ECG was done in about 30% of the time, and third was chest pain with initial normal ECG. In such cases, we found that cases of ACS would not have been picked if no repeated ECG was done. That is why we have enforced a policy of repeated ECG if patients remain symptomatic and a high index of suspension for STEMI is contemplated. This was present in 10% of the cases. There are also other causes: ~30% related to specific cases like the one presenting with nondiagnostic ECG (ie, old changes of established MI vs. new changes), or suspected other critical diagnoses like pulmonary embolism or aortic dissection or severe underlying comorbid conditions that will preclude early invasive strategy. In such cases, we enforce early communication to seek expert opinion with interventionalists on duty to get early consensus with a clear plan for appropriate management. Finally, in other cases of ACS, there was reduced length of stay to 2.5 days compared with 4 days secondary to early invasive strategy and detection of cases. It is worth mentioning that the effect was continuous in the fourth quarter on both KPIs. We intentionally reported the data of 6-month protocol implementation to answer the question of “how long does it take for this protocol to work, if it is going to work?”

## CONCLUSIONS

This short-time trial, despite its limitations of being conducted at a single center and being open labeled with no control group, has demonstrated clearly that following CPPs with continuous and close monitoring using a smart phone application could improve KPIs related to ACS patient, such as door-to-first-ECG and door-to-needle time. There is a strong correlation between compliance rate with pathways and targeted areas of improvement.

**TABLE 1.** Patients’ Characteristics

Patient Characteristic	n	%
Mean age	58 ± 15	
Total cases of chest pains	2067 total 1610 noncardiac pain 457 ACS 63 STEMI	77% 22% 3%
Gender	Male = 319/457 Female = 137/457	70% 30%
Risk factor for atherosclerotic heart disease	Diabetes 206/457 Hypertension 283/457 Hyperlipidemia 178/457 Family history of CAD 50/457 Smoking 85/457	45% 62.7% 39.1% 11.6% 18.7%

## DISCLOSURES

*None declared.*

## Supplementary Material


