# Supplementary material for: Implementing “Chest Pain Pathway” Using Smartphone Messaging Application “WhatsApp” as a Corrective Action Plan to Improve Ischemia Time in “ST-Elevation Myocardial Infarction” in Primary PCI Capable Center “WhatsApp-STEMI Trial”
Source: Crit Pathw Cardiol. 2021 Nov 23;20(4):179–84. doi: 10.1097/HPC.0000000000000264 (PMC8603662; doi:10.1097/HPC.0000000000000264)
Supplement: Supplementary file 1 [file hpc-20-179-s001.pdf]

DR SULAIMAN AL HABIB medical group	HMG/TAK/CCU/CP/001
	Issue Date: 07/01/2020
	Effective Date: 14/01/2020
CORONARY CARE UNIT	Review Date: 13/01/2021
Title: Clinical Pathway of Chest Pain Management	

Always Use Clinical Judgment

Triage Date / Time: **Time of Onset of Chest Pain:**   **Triage Category**:
Vitals: Temp: HR: RR: BP: SPO2:   Nurse Signature: Nurse ID:

Chest Pain or Symptoms of Myocardial Ischemia
(Triage Level 2)

Please tick:
[ ] Sweating [ ] Syncope [ ] Dyspnea [ ] Epigastric discomfort [ ] jaw pain [ ] Sudden orthopnea [ ] arm pain
Be aware of a Typical presentations in [ ] Diabetics, [ ] Renal Failure, [ ] Female, [ ] Elderly or [ ] Aboriginal

Always Consider:
Aortic Dissection: [ ] Back pain, [ ] Hypertension, [ ] Absent Pulse, [ ] BP Difference
Pulmonary Embolism: [ ] Severe dyspnea, [ ] respiratory distress, [ ] Low O2 saturation

ECG + Vital Signs + Expert Interpretation
Within 10 minutes
Review Time:
Right sided ECG (V4R) if inferior ST-elevation present
****Contact Cardiology service if ECG advice required.**

Pulmonary Embolism

Increase RR & HR
ECG changes:
Sinus tachy; RAD; RBBB; S1Q3T3

If History; Physical Exam; ECG changes suggestive of PE
EXIT PATHWAY
And commence Venous Thromboembolism Pathway

NON-ACS cause of chest pain identified:

Exit Pathway

Acute Coronary Syndrome

General Management (Please Tick):

[ ] **Aspirin.**
[ ] **Nitrates** (I/V OR S/L).
[ ] **IV Access** x 2.
[ ] **Labs**: Cardiac Troponin; CBC, Kidney Profile; Blood glucose; INR.
[ ] **Pain Relief** (Morphine for persistent pain).
[ ] **Continuous Cardiac Monitoring.**
[ ] **Oxygen** if SPO2 <93%
[ ] **Chest X-Ray.**
[ ] Frequent Observation.
[ ] **Repeat ECG** for persistent Chest Pain.
[ ] **Beta Blockers if indicated.**
[ ] **Mild tranquilizer** (usually a benzodiazepine) in case of extreme patient anxiety.

St Elevation or (Presumed New) Bundle Branch Block

NO → NSTEMI Pathway on **Page 4**

YES → ER Consultant To Call 111 and **Activate** **CODE STEMI**
TIME:
Then (Go To **Page 2** of the pathway)

ER Consultant Date/Time & Stamp

This Page to Be Completed by **ER Physician**

CCU Specialist

1. **Confirm Indication for re-perfusion:**

Chest Pain >30min and <12hours.
Persistent ST elevation ≥1mm in 2 contiguous limb leads or persistent ST elevation in ≥2mm in 2 contiguous chest leads or new or presumed new LBBB
Myocardial Infarct likely from history.

2. **Choose reperfusion therapy:**

Primary PCI Unless

[ ] Significant delay to availability of Cath Lab.
[ ] Patient does not consent to PCI.
[ ] History, Contrast Allergy.
[ ] Vascular Access Problems.

Thrombolysis (If appropriate)

Exit Pathway
And commence Thrombolysis for STEMI pathway
(Page 3)

Discuss with Interventional Cardiologist:
Time:

Decision regarding re-perfusion therapy:
Time:
[ ] Activate Cathlab Team through nursing supervisor.
[ ] Explain the procedure to patient and family.
[ ] Secure procedure, Anesthesia and High Risk Consent.

3. Administer Antithrombotic Therapy:		√	X
Aspirin	300mg (Soluble) [Loading]		
Clopidogrel	300-600mg (Loading)		
or			
Ticagrelor	180 mg (Loading)		
Enoxaparen 0.5mg/kg i.v bolus			
or			
Heparin 70-100 IU/kg i.v Bolus			

Other Code STEMI Team Members
Immediately report to the location of STEMI

Below To Be Filled By ER Nursing Team Leader:

NAME

Duty Manager:
☐ ID: …………… Time of Report: ......:......
☐ Check the eligibility and clearance of the Patient.
☐ Settle Insurance concerns.
☐ Settle Admission concerns.

NAME

Nursing Supervisor:
☐ ID: …………… Time of Report: ......:......
☐ Confirm the Cath LAB Activation.
☐ ***If CCL Team is out of duty hours,
☐ Call the Transportation Department and arrange transportation for Cath-LAB Staff pick up.
☐ Provide updates to Cath-LAB on call Team Leader.

NAME

Cath LAB Team Leader:
☐ ID: …………… Time of Report: ......:......
☐ Immediately Responds to Call.
☐ Coordinates with Nursing Supervisor.
☐ Activates the Cath-LAB Team Members.
☐ Provide updates to Cath-LAB Team.
☐ Informs the Emergency Department, CCL Team arrival and if Patient can be shifted to the Cathlab.

NAME

Anesthesiologist:
☐ ID: …………… Time of Report: ......:......
☐ Immediately Responds to Call.
☐ Standby if needed.

NAME

CCU Team Leader / Charge Nurse:
☐ ID: …………… Time of Report: ......:......
☐ Accompany patient from ER to Cath-LAB.
☐ Check completeness of patient documents.
☐ Arrange bed with CCU for post procedure.

Cath-LAB Arrival Time: ………..
On Table Time: ………..
First Device Use Time: ………..

Fibrinolytic Therapy Pathway

Absolute – NOT for treatment	Yes	No
Patient is currently taking Warfarin (if YES – SEE BELOW)		
• **INR >2.0** consider risk/benefit. SEEK EXPERT ADVICE		
Patient taking a new oral anticoagulant e.g. Dabigatran. SEEK EXPERT ADVICE		
Recent major surgery, trauma, head injury within **3 weeks**?		
Recent stroke within **6 months**?		
G.I. Bleed within **1 month**?		
Haemorrhagic diathesis?		
Aortic dissection?		

**Relative. If yes to any below, consider RISK/BENEFIT, SEEK EXPERT ADVICE**	Yes	No
>80 years inferior MI with minimal ST elevation without ST depression		
Blood pressure ≥180mmHg systolic, ≥100mmHg diastolic?		
Prolonged chest compression?		
Active peptic ulcer?		
Other significant risk of haemorrhage?		
Pregnant or post partum 1 week?		

Administration of Fibrinolytic Therapy

**Body Weight = _____kg**			**Prescriber Time/Date**	**Given by Time/Date**
**Drug**	Initial Treatment (Dose)	Given		
**Alteplase**	15mg i.v Bolus<br>0.75mg/kg i.v over 30min (upto 50mg) then 0.5mg/kg i.v over 60 min (upto 35mg)	☐		
Or				
**Tenectaplase**	Single i.v bolus (10 seconds):<br><60kg = 30mg (6000 IU)<br>60-69kg = 35mg (7000 IU)<br>70-79kg = 40mg (8000 IU)<br>80-89kg = 45mg (9000 IU)<br>> 90kg = 50mg (10,000 IU)<br>It is recommended to reduce to ½ dose if age > 75 yr	☐		
**Enoxaparin**	<75 years: 30 mg i.v. bolus followed 15 minutes later by 1mg/kg s.c every 12 hours until revascularization or hospital discharge for a maximum of 8 days. The 1st two doses should not exceed 100mg per injection.	☐		
Or				
**UFH**	60 IU/kg i.v. bolus with a max of 4000 IU followed by an i.v. infusion of 12 IU/kg with a max of 1000 IU/hr for 24-48 hours. Target aPTT 50-70 seconds or 1.5-2.0 times that of control to be monitored at 3,6,12 an 24 hours.	☐		

Ensure the patient is prescribed the following anti-platelet treatment:
[ ] **Aspirin** 300mg, oral, stat, then 75mg, po, daily and [ ] **Clopidogrel** 300mg, oral, stat immediately, then 75mg, oral, daily. ***Do not give Ticagrelor before or after Fibrinolytic use.***

[ ] **Failure**, after 90 minutes of receiving Fibrinolytic, to reduce ST-segment elevation (in lead showing maximum ST-elevation pre-thrombolysis) at least 50%.
[ ] **Within 12 hours** of onset of chest pain [ ] **Patient fit and willing** to transfer and undergo PCI

→ **Discuss Rescue PCI**

This Side to be Completed by ER Physician

NSTEMI - ACS Pathway

Grace Score:	

High Risk Features - Clinical features consistent with ACS and any of the following:

[ ] Repetitive or prolonged (>10 mins) ongoing chest pain or discomfort.
[ ] Elevated Troponin.
[ ] Persistent or dynamic ECG changes of ST segment depression ≥0.5mm or new T-wave inversion ≥2mm.
[ ] Transient ST-segment elevation (≥0.5mm) in more than two contiguous leads.
[ ] Hemodynamic compromise - systolic blood pressure <90mmHg, cool peripheries, diaphoresis, Killip Class >1, and / or new-onset mitral regurgitation.
[ ] Sustained ventricular tachycardia.
[ ] Syncope.
[ ] Left ventricular systolic dysfunction (left ventricular ejection fraction <0.40), and / or clinical evidence of heart failure.
[ ] Prior percutaneous coronary intervention within 6 months or prior coronary artery bypass surgery.

Yes to Any →

NO TO ALL ↓

Intermediate / Low Risk Features - Clinical features consistent with ACS and any of the following:

[ ] Resolved chest pain or discomfort within the past 48 hours that occurred at rest, or was repetitive or prolonged (>10 min).
[ ] Age >65 years.
[ ] Diabetes with typical or atypical symptoms of ACS.
[ ] Chronic kidney disease (GFR <60 mL / minute) with typical or atypical symptoms of ACS).
[ ] Known Coronary Artery Disease (CAD) or previous Myocardial Infarction (MI).
[ ] Two or more of the following risk factors: known hypertension, family history, active smoking or hyperlipidemia.
[ ] Prior regular aspirin use.
[ ] Recent onset of crescendo or unstable angina symptoms.

Yes to Any →

↓ **NO TO**

Refer To Page 5-P1 for Discharge Algorithm (ER Physicians)

This Side to be Completed by Cardiology Specialist

High Risk NSTE-ACS (TIMI Risk Score 5-7)

[ ] Admit to CCU/ICU/HDU; commence ACS pathway.
[ ] Continuous Cardiac Monitoring and frequent vital signs monitoring.
[ ] Repeat ECG immediately if symptoms recur.
[ ] Repeat ECG 8 hrs. post onset of symptoms.
[ ] Repeat Troponin at 8 hrs. if 1st sample negative.
[ ] ECG/Troponin review by ER specialist or Cardio Specialist.

Medications:

[ ] **Aspirin** 300mgs stat. followed by 81mgs once daily.
[ ] **GTN** 2-10 mg/hr IV infusion (titrate to pain and BP).
[ ] **Bisoprolol** 2.5mg-5.0mg daily if not C/I.

[ ] **Clopidogrel** 300mgs stat than 75mgs once daily.
or
[ ] **Ticagrelor** 180mg Loading dose, than 90mg per Os, twice daily.

[ ] **Fondaparinux** 2.5 mg S.C. daily.
or
[ ] **Enoxaparin** 1mg/kg S.C. twice a day.
or
[ ] **Heparin** 60–70 IU/kg Bolus (MAX 5000 IU) then infusion per protocol.

Re-Stratify Risk:
Admit to:
[ ] Regular vital observations.
[ ] Repeat ECG and Troponin at 3–6 hours (OR 6–8 hours for point-of-care test).
[ ] Does not require continuous cardiac monitoring if first (0 hour) Troponin negative, ECG normal, and no further chest pain

Manage as High Risk if:
[ ] Positive Troponin.
[ ] New ECG changes.
[ ] Recurrent chest pain or develops other high risk features.

If TIMI Risk Score 0:
[ ] Discharge and refer for OPD Exercise Stress Test (EST) or, alternative testing within 14 days.

If TIMI Risk Score 1-4:
Refer for urgent in-patient EST or alternative testing .

TIMI risk score (Add up ticks)	

TIMI Risk Score:
[ ] Age ≥ 65 Years.
[ ] ≥ CAD risk factors.
[ ] Known CAD (Stenosis 50%).
[ ] ASA use in past 7 days.
[ ] Recent (≤24hrs) severe angina.
[ ] Troponin ST deviation ≥0.5mm.

Refer to Page 6- P1 for Invasive Management for NSTEMI

DR SULAIMAN AL HABIB medical group	HMG/TAK/CCU/CP/001
	Issue Date: 07/01/2020
	Effective Date: 14/01/2020
CORONARY CARE UNIT	Review Date: 13/01/2021
Title: Clinical Pathway of Chest Pain Management	

PAGE 5 - P1 – 0 HR / 3 HR, NSTE-ACS Rule Out Algorithm (For ER Physicians)

Please Tick the Boxes

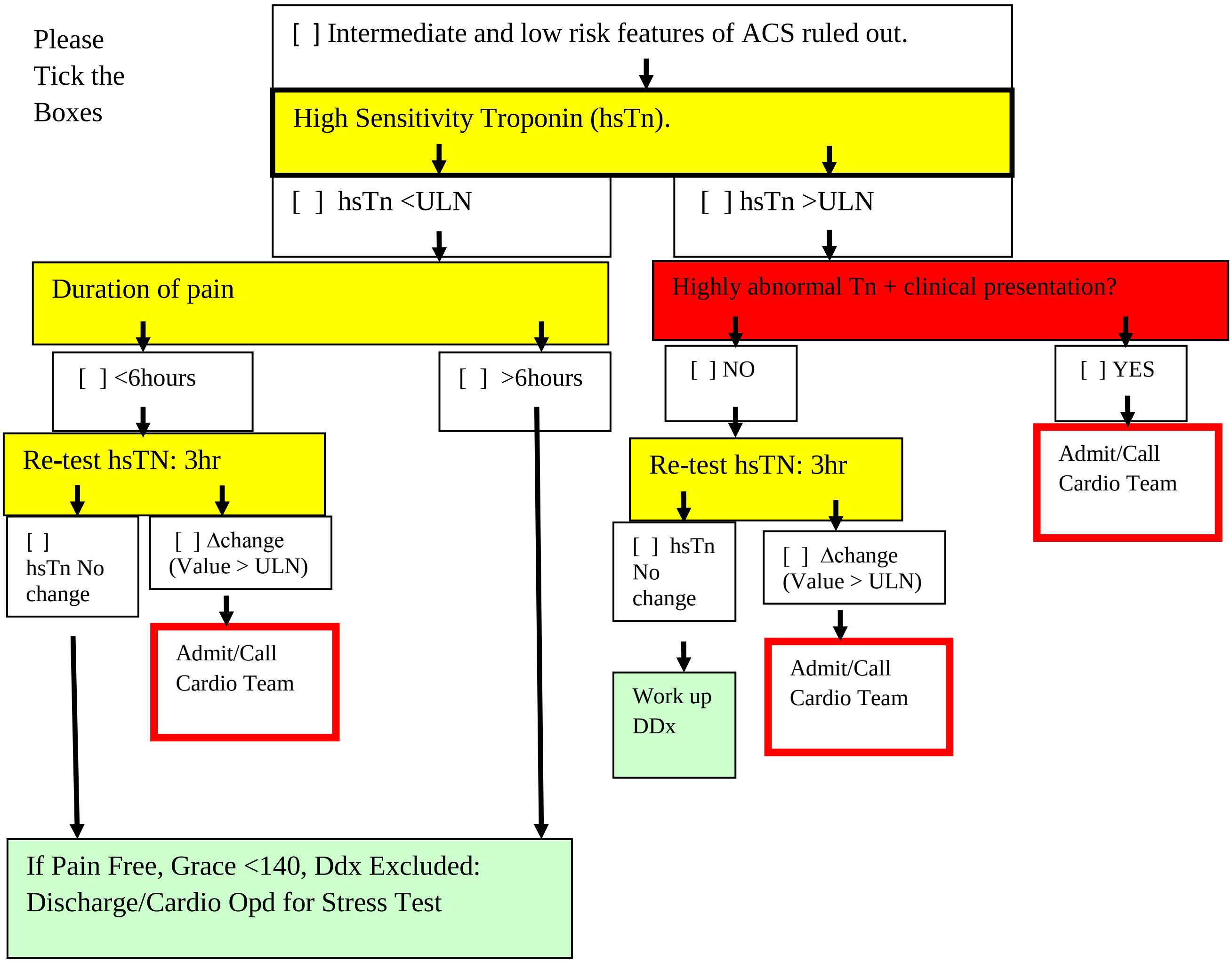

ER Consultant Date/Time & Stamp

KEY:

hsTn: High sensitivity cardiac troponins.

ULN: Upper Limit Normal.

*** **Reference: European Society of Cardiology**.

PAGE 6 - P1 - Risk Criteria for Invasive Management (For Cardiology Team)

Very High Risk (Immediate Invasive <2hr)

[ ] Haemodynamic instability or cardiogenic shock.
[ ] Recurrent or ongoing chest pain refractory to medical treatment.
[ ] Life-threatening arrhythmias or cardiac arrest.
[ ] Mechanical complications of MI.
[ ] Acute heart failure.
[ ] Recurrent dynamic ST-T wave changes, particularly with intermittent ST-elevation.

High Risk (Early Invasive <24HR)

[ ] Rise or fall in cardiac troponin compatible with MI.
[ ] Dynamic ST- or T-wave changes (symptomatic or silent).
[ ] GRACE score >140.

Intermediate Risk (Invasive <72 HR)

[ ] Diabetes mellitus.
[ ] LVEF <40% or congestive heart failure.
[ ] Early post-infarction angina.
[ ] Prior PCI.
[ ] Prior CABG.
[ ] GRACE risk score >109 and < 140.

Secondary Prevention Discharge Medications

**Secondary Preventative Discharge Drugs / Advice**	**Yes**	**No**	**Contra-indicated (State Reason)**
Life Style: Smoking Cessation; Regular Physical activity; Healthy Diet.	☐	☐	
Aspirin 81mg, per os, once daily.	☐	☐	
Ticagrelor (Brilinta) 90 mg, per os, twice daily, **or**<br>Plavix 75mg, per os, once daily.	☐<br>☐	☐<br>☐	
Atorvastatin 80mg, per os, once daily (> 80 years: 40mg) for 6 months.	☐	☐	
ACE Inhibitor (Especially if LVEF > 40%, Heart Failure; HTN; DM).	☐	☐	
Beta-Blocker (Especially if LVEF > 40%).	☐	☐	
Mineralocorticoid receptor antagonist: (i.e. Eplerenone; especially with LVEF > 35%; HF or DM after NSTE-ACS).	☐	☐	
Cardiac Rehabilitation Program.	☐	☐	

2.0 Reference:

2.1 European Society of Cardiology.

2.2 2017 ESC Guidelines for the management of acute myocardial infarction in patients presenting with ST-segment elevation European Heart Journal (2018) 39, 119–177 doi:10.1093/eurheartj/ehx393.

2.3 2015 ESC Guidelines for the management of acute coronary syndromes in patients presenting without persistent ST-segment elevation European Heart Journal (2018) 39, 119–177 doi:10.1093/eurheartj/ehx393.

2.4 Possible Cardiac Chest Pain Clinical Pathway; Queensland Health, 2017.

2.5 NICE: Myocardial infarction with ST-segment elevation: acute management.

2.6 Joint Commission International Accreditation 6th Edition, 2017.

2.7 Central Board for Accreditation of HealthCare Institution 3rd Edition, 2015.

3.0 Distribution:

3.1 General Director.
3.2 Executive Director.
3.3 Medical Director.
3.4 Director of Nursing.
3.5 Quality Manager.
3.6 All Clinical HOD.
3.7 Medical Admin Office.
3.8 Quality Improvement Department.

4.0 Revision History:

Revision No.	Date	Original Clause No.	Revised Clause No.	Remarks
Revision. No. 1	Jan. 07, 2020	-----	-----	New Clinical Pathway

APPROVAL BLOCK		
Prepared By		Date
	Cardiology Consultant	07-01-20
Reviewed By		Date
	Medical Administration	08-01-20
Reviewed By		Date
	Cardiology HOD	08-01-20
Reviewed By		Date
	Quality Manager	09-01-20
Reviewed By		Date
	Director of Nursing	09-01-20
Reviewed By		Date
	Medical Director	12-01-20
Reviewed By		Date
	Executive Director	13-01-20
Approved By		Date
	General Director	14-01-20

Accreditation/ Certification	JCIA	CBAHI	
Meets the standard/ measurable element of	√	√	
